# Single-cell RNA sequencing unveils macrophage heterogeneity and cell-cell interactions in hepatocellular carcinoma progression

**DOI:** 10.3389/fgene.2026.1819908

**Published:** 2026-05-18

**Authors:** YiFei Tang, JiaHui Li, LingYing Huang

**Affiliations:** 1 Department of Liver Diseases, ShuGuang Hospital Affiliated to Shanghai University of Chinese Traditional Medicine, Shanghai, China; 2 Department of TCM Gynecology, The International Peace Maternity and Child Health Hospital, School of Medicine, Shanghai Jiao Tong University, Shanghai, China

**Keywords:** APOA2, epigenetic regulation, hepatocellular carcinoma, intercellular communication, macrophage heterogeneity, single-cell RNA sequencing, tumor microenvironment

## Abstract

**Background:**

Hepatocellular carcinoma (HCC) represents a major global health burden, characterized by complex metabolic reprogramming and immunological dysregulation. This study aimed to elucidate the molecular mechanisms underlying HCC progression using integrative multi-omics analyses, with a specific focus on macrophage heterogeneity and intercellular communication networks in the tumor microenvironment.

**Methods:**

We performed comprehensive bioinformatic analyses integrating gene expression profiling, DNA methylation data, and single-cell RNA sequencing (scRNA-seq) datasets from publicly available databases. Single-cell transcriptomic data (GSE149614) were processed using Seurat for quality control, dimensionality reduction, and cell type annotation. Macrophage subpopulation diversity was assessed through Weighted Gene Co-expression Network Analysis (WGCNA) and differential expression analysis. Intercellular communication networks were reconstructed using CellPhoneDB and CellChat to identify Signaling axes that act as primary mediators of macrophage–hepatocyte/fibroblast crosstalk in HCC. Functional enrichment analyses were conducted via Gene Ontology and KEGG pathway analyses. The diagnostic and prognostic potential of genes whose expression or methylation status predicts HCC stage, metastasis risk, and patient survival was evaluated through Receiver Operating Characteristic curve analysis and survival modeling. Expression patterns of genes whose dysregulation directly disrupts immunometabolic crosstalk between macrophages and tumor cells, whose dysregulation increases the risk of hepatocellular carcinoma progression by promoting an immunosuppressive microenvironment and enhancing tumor cell proliferation and invasion were experimentally validated using quantitative real-time PCR (qRT-PCR) in two hepatocellular carcinoma cell lines (HepG2 and Huh7) obtained from American Type Culture Collection.

**Results:**

Single-cell analysis revealed profound cellular heterogeneity within the HCC microenvironment, identifying six transcriptionally distinct macrophage subpopulations (M1–M6) with unique immunometabolic signatures. M2-like subsets were enriched in extracellular matrix organization and integrin-mediated signaling pathways, supporting their pro-fibrotic and immunosuppressive roles. Intercellular communication network analysis identified the SPP1–CD44/ITGAV signaling axis as a dominant pathway mediating macrophage–hepatocyte and macrophage–fibroblast interactions. APOA2 demonstrated differential expression between normal and tumor tissues, with its downregulation strongly correlated with promoter methylation (Spearman ρ = −0.31, P = 9.11 × 10^−10^). Although APOA2 showed limited diagnostic performance (AUC = 0.552), APOA2-low tumors exhibited trends toward poorer clinical outcomes across multiple survival metrics. Integration of metabolomic and transcriptomic data revealed associations between APOA2 silencing, altered serum metabolite profiles, and enhanced macrophage activation, establishing a metabolic–immune–epigenetic cascade that promotes tumor fibrogenesis and progression. qRT-PCR validation in HCC cell lines confirmed differential expression of Genes encoding core components of the SPP1–CD44/ITGAV signaling axis that regulate macrophage–tumor crosstalk and whose expression levels correlate with higher histological grade and increased metastatic risk in hepatocellular carcinoma in aggressive Huh7 cells (3.2-fold and 2.8-fold respectively, P < 0.001), while APOA2 was downregulated (0.35-fold, P < 0.001), corroborating bioinformatic predictions.

**Conclusion:**

This study unveils Macrophages that orchestrate the majority of intercellular signaling interactions in the HCC microenvironment orchestrating the immunometabolic landscape of HCC through the SPP1–integrin signaling network. The identification of functionally distinct macrophage subpopulations and the metabolic–immune–epigenetic axis involving APOA2 provides novel mechanistic insights into HCC pathogenesis and identifies genes and signaling axes whose pharmacological inhibition can reverse immunosuppression and block HCC progression for precision intervention strategies.

## 1 Introduction

Over the past decades, alcohol-related liver disease, especially alcoholic liver fibrosis and cirrhosis, has emerged as a major global health burden. Among them, alcohol-related hepatocellular carcinoma (HCC) is one of the most severe consequences of alcohol-related liver cancer. Despite some progress in HCC research, its pathogenic mechanisms remain incompletely understood. In recent years, increasing evidence suggests that serum metabolites and immune cells play crucial roles in the development of alcohol-related liver cancer (Cheng et al., 2025; Wang et al., 2025; Zhao et al., 2025).

Serum metabolites during alcohol related liver cancer is being recognized. Chronic and high-dose alcohol consumption causes metabolic disorders including fatty acid, amino acid, and glucose metabolism disorders. These metabolic dysregulations not only intrinsically injure the liver but also disrupt the homeostasis and function of immune cells. Dysregulation of fatty acid metabolism, for instance, causes fat to be deposited in the liver where it causes steatosis, which is a vital step in the progression of alcohol-related liver damage. Disturbances in amino acid metabolism can modify the proliferation and differentiation of immune cells and, in turn, modulate immune responses (Li et al., 2023; Zhao et al., 2024; Zou et al., 2023).

Immune cells play important roles in alcohol-related liver cancer as well. Long-term intake of alcohol and in excess alters the regular activity of the immune system which increase inflammatory responses and oxidative stress that damages liver even more. For example, a change in this ratio in the activity of T lymphocytes and macrophages is often found in drinkers and is closely associated with the severity of alcohol-related liver cancer. Moreover, alcohol has an influence on the maturation and functionality of dendritic cells leading to a skewed immune response (Chu et al., 2024; Ning et al., 2024; Nov et al., 2024).

The interaction of immune cells and serum metabolites contribute to the pathogenesis of alcohol-related liver cancer development. As one example, levels of specific serum metabolites can alter the activity and function of immune cells, and the activity and function of immune cells can modify the synthesis and degradation of metabolites. This reciprocal impact could create a complicated feedback loop that additionally favors the development of alcohol-related liver cancer (Jiao et al., 2024; Jung et al., 2023; Koelsch et al., 2023).

These findings imply that serum metabolites and immune cells are essential participants in the formation of alcohol-related liver cancer. Elucidating these mechanisms will not only help us to understand better the etiology of HCC but will offer novel insights and approaches for its prevention and treatment. Further studies should focused on the mechanisms of interaction between serum metabolites and immune cells, and more importantly, how this interaction promotes the development of alcohol related liver cancer. Further clinical trials are also necessary to confirm these hypotheses and provide dependable evidence contributing to the introduction of novel treatment methods.

## 2 Materials and methods

### 2.1 Complementary study design and data acquisition

This study further integrated multi-omics data and bioinformatics approaches to comprehensively elucidate the molecular characteristics and cellular landscape of hepatocellular carcinoma (HCC). These supplementary analyses primarily focused on the expression, methylation, and alternative splicing patterns of specific genes (e.g., APOA2) in HCC, as well as the heterogeneity of immune cells (particularly macrophages) within the tumor microenvironment and their potential associations with HCC progression. Data were mainly obtained from public high-throughput sequencing datasets, including gene expression profiling (RNA-seq), DNA methylation profiling, and single-cell RNA sequencing (scRNA-seq) data, ensuring broad representativeness and reliability of the research findings.

### 2.2 Single-cell RNA sequencing analysis

Single-cell transcriptomic data (GSE149614) were obtained from the Gene Expression Omnibus (GEO) database to characterize the cellular heterogeneity of hepatocellular carcinoma (HCC). Data preprocessing and quality control were performed using the Seurat (v4.3.0) R package. Cells with fewer than 200 detected genes or >10% mitochondrial gene expression were excluded. Gene expression matrices were normalized and log-transformed. Highly variable genes were identified and used for dimensionality reduction via principal component analysis (PCA), followed by clustering using Uniform Manifold Approximation and Projection (UMAP) and t-distributed Stochastic Neighbor Embedding (t-SNE).

Cell type annotation was achieved by comparing cluster-specific marker genes with known cell type–specific signatures using SingleR and manual curation based on canonical markers (e.g., CD68, SPP1, CD44 for macrophages). To assess macrophage subpopulation diversity, Weighted Gene Co-expression Network Analysis (WGCNA) and differential expression analysis were conducted, identifying six distinct macrophage subsets (M1–M6). Intercellular communication networks were reconstructed using CellPhoneDB and CellChat, focusing on the SPP1–CD44/ITGAV signaling axis that mediates macrophage–hepatocyte and macrophage–fibroblast interactions.

Functional enrichment of macrophage subsets was performed via Gene Ontology (GO) and Kyoto Encyclopedia of Genes and Genomes (KEGG) analyses, revealing enrichment in extracellular matrix organization and integrin-mediated signaling pathways. Visualization and statistical analyses were conducted using R (v4.3.2) and Python (v3.9).

### 2.3 Cell culture and quantitative real-time PCR validation

To validate the expression patterns of Genes that define macrophage subpopulation identity, shape intercellular communication networks, and modulate APOA2-dependent lipid metabolism and epigenetic silencing in hepatocellular carcinoma identified from single-cell RNA sequencing analysis, two human hepatocellular carcinoma cell lines were obtained from the American Type Culture Collection (ATCC): HepG2 (ATCC HB-8065) and Huh7 (JCRB0403). HepG2 cells represent a well-differentiated hepatocellular carcinoma line with epithelial morphology, while Huh7 cells display a more poorly differentiated phenotype with mesenchymal characteristics. Both cell lines were maintained in Dulbecco’s Modified Eagle Medium (DMEM, Gibco) supplemented with 10% fetal bovine serum (FBS, Gibco), 100 U/mL penicillin, and 100 μg/mL streptomycin (Gibco) at 37 °C in a humidified atmosphere containing 5% CO2. Cells were passaged every 2–3 days upon reaching 80% confluence and were used between passages 5–20 for all experiments.

For qRT-PCR validation, total RNA was extracted from cultured cells using TRIzol reagent (Invitrogen, Carlsbad, CA) according to the manufacturer’s protocol. RNA concentration and purity were assessed using a NanoDrop 2000 spectrophotometer (Thermo Scientific), with A260/A280 ratios between 1.8 and 2.0 considered acceptable. First-strand cDNA synthesis was performed using 1 μg of total RNA with the PrimeScript RT Reagent Kit (Takara Bio, Shiga, Japan) following the manufacturer’s instructions. Quantitative RT-PCR was performed on a QuantStudio 5 Real-Time PCR System (Applied Biosystems) using SYBR Green PCR Master Mix (Applied Biosystems). Each 20 μL reaction contained 10 μL SYBR Green Master Mix, 0.4 μL each of forward and reverse primers (10 μM), 2 μL cDNA template, and 7.2 μL nuclease-free water. The thermal cycling conditions were: initial denaturation at 95 °C for 30 s, followed by 40 cycles of 95 °C for 5 s and 60 °C for 34 s, with a final melting curve analysis to verify amplicon specificity.

Gene-specific primers were designed using Primer-BLAST (NCBI) and synthesized by Integrated DNA Technologies. The primer sequences were as follows: SPP1 forward 5′-CTC​CAT​TGA​CTC​GAA​CGA​CTC-3′, reverse 5′-CAG​GTC​TGC​GAA​ACT​TCT​TAG​AT-3’; CD44 forward 5′-CTG​CCG​CTT​TGC​AGG​TGT​A-3′, reverse 5′-CAT​TGT​GGG​CAA​GGT​GCT​ATT-3’; APOA2 forward 5′-CTG​AAG​GAT​GCC​TAC​GAG​AAG-3′, reverse 5′-CAG​CAT​CAC​AGT​CAC​TGT​CC-3’; CD68 forward 5′-GCT​ACA​TGG​CGG​TGG​AGT​ACA​A-3′, reverse 5′-ATG​ATG​AGA​GGC​AGC​AAG​ATG​G-3’; GAPDH forward 5′-GAG​TCA​ACG​GAT​TTG​GTC​GT-3′, reverse 5′-TTG​ATT​TTG​GAG​GGA​TCT​CG-3’. All reactions were performed in technical triplicates, and three independent biological replicates were conducted for each cell line. Relative gene expression was calculated using the 2^−^ΔΔCt method with GAPDH as the endogenous reference gene. Statistical significance was assessed using unpaired two-tailed Student’s t-tests, with P-values less than 0.05 considered statistically significant.

### 2.4 Bioinformatic and statistical analyses

For data processing and analysis, a variety of bioinformatics tools and statistical methods were employed. Gene expression analysis involved evaluating differential gene expression between normal and tumor tissues, utilizing Receiver Operating Characteristic (ROC) curves and calibration curves to assess the diagnostic efficacy of potential biomarkers. DNA methylation analysis focused on identifying CpG sites whose methylation levels show statistically significant correlations with cognate gene expression levels and investigating their methylation patterns in HCC. Furthermore, Weighted Gene Co-expression Network Analysis (WGCNA) was conducted to identify gene modules correlated with specific cell types or disease states. Cell type annotation and cell-cell interaction analyses were performed based on single-cell sequencing data, involving dimensionality reduction algorithms (e.g., UMAP and t-SNE) for clustering and visualization of cell populations, and the construction of cell-cell communication networks. Finally, survival analyses (including overall survival, disease-free survival, etc.) were performed to evaluate the impact of genes with prognostic relevance and functional significance in HCC progression or molecular features on HCC patient prognosis.

### 2.5 Statistical analyses

Here, through a Two-Sample Mendelian Randomization (TSMR) analysis approach, we systematically catalog the causal relationships among immune cells, serum metabolites, and HCC. In case of exposure factors having multiple IVs, the study adopted the IVW method with multiplicative random effects since it is one of the most efficient methods for the calculation of causal effects while allowing heterogeneity of the causal estimates. The Wald ratio analysis method was applied to obtain the estimates of effect for single (and multiple) instrumental variables on exposure factors.

For blood metabolites of each class, a dynamic threshold of P < 0.05/n for blood metabolites, where n is the number of blood metabolites with valid IVs in the corresponding class, was set up in the analysis of statistics. Using multiple testing significance thresholds, the study divided results into significant causal associations that met the threshold, and potential causal associations with p-values <0.05 but did not meet the primary threshold. These possible associations indicated trends in causative links, but they should be further addressed to establish significance.

To ensure the robustness of research findings, the research team implemented a multi-layered validation strategy. Initially, the heterogeneity of causal inferences was assessed by calculating the Cochran’s Q statistic. Subsequently, MR Egger regression and weighted median methods were employed to rigorously evaluate both significant and potential causal associations. Furthermore, the study conducted in-depth analyses of TSMR results with ≥2 results that passed sensitivity analysis. Additionally, the research performed Mendelian randomization analyses on multiple exposure combinations using weighted linear regression models similar to the IVW method (Bowden, 2017; Rees et al., 2017).

This multi-dimensional, systematic analytical approach not only enhanced the scientific rigor of the research but also provided an innovative research paradigm for unveiling the complex mechanisms of hepatocellular carcinoma development. By integrating analytical perspectives from immunology, metabolomics, and genetics, the study aimed to comprehensively understand the molecular-level mechanisms of HCC progression, charting new directions for precision medicine research. The significance of the study extends beyond its methodological innovation, offering a novel scientific explanatory framework for understanding HCC occurrence and development, and establishing crucial theoretical foundations for potential individualized prevention and treatment strategies.

## 3 Results

### 3.1 APOA2 expression and diagnostic potential in hepatocellular carcinoma and macrophage heterogeneity

**FIGURE 1:**
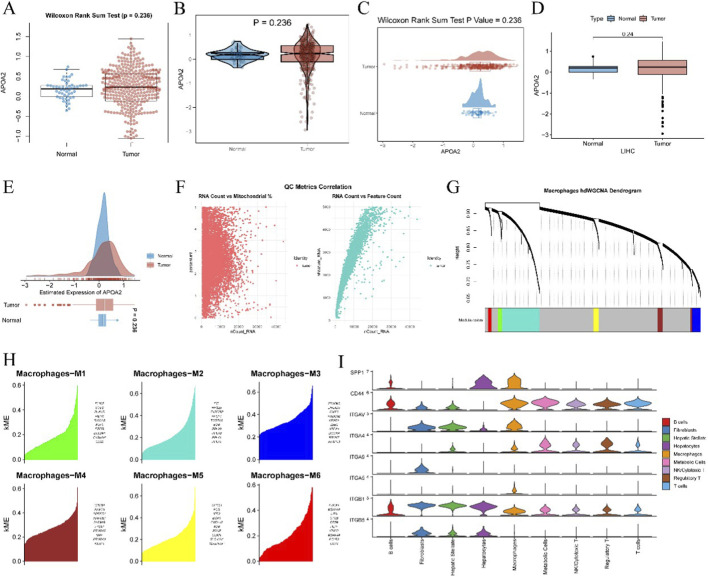
Analysis of APOA2 Expression and Macrophage Subtypes in Liver Cancer. **(A)** Box plot illustrating APOA2 expression levels in normal liver tissue versus hepatocellular carcinoma (HCC) tumor tissue, showing statistical differences. **(B)** Violin plot depicting the distribution density and median of APOA2 expression in normal and tumor samples. **(C)** Combined violin and scatter plot showing the expression distribution of APOA2 in normal and tumor tissues. **(D)** Box plot comparing APOA2 levels in normal liver tissue and Liver Hepatocellular Carcinoma (LIHC) tumor samples. **(E)** Density plot illustrating the estimated expression distribution of APOA2 in normal and tumor tissues. **(F)** Quality control metrics for RNA sequencing data, with the left panel showing RNA count versus mitochondrial percentage, and the right panel showing RNA count versus feature count, used to assess data quality. **(G)** Dendrogram illustrating the clustering of macrophage modules, likely derived from Weighted Gene Co-expression Network Analysis (WGCNA). **(H)** Bar plots displaying the module eigengenes (KME) for different macrophage subtypes (M1-M6), reflecting gene expression patterns within each subtype. **(I)** Violin plots showing the estimated expression of genes (CD44, ITGAV, ITGA4, ITGA5, ITGB1) across various annotated cell types, including B cells, Fibroblasts, Hepatic Stellate cells, Hepatocytes, Macrophages, Metabolic Cells, NK/Cytotoxic T cells, Regulatory T cells, and T cells.

Figure 1A shows the expression distribution of APOA2 in normal and tumor tissues (Wilcoxon rank-sum test, *P* = 0.236). Figures 1B,C further illustrate the expression distribution of APOA2 in normal and tumor tissues using violin plots, both with *P* = 0.236. Figure 1D, a box plot, also displays APOA2 expression in LIHC normal and tumor tissues, with a p-value of 0.24. Figure 1E presents the estimated expression of APOA2 in normal and tumor tissues as a density plot, showing a p-value of 0.236. Figure 1F displays two quality control metrics correlation scatter plots: “RNA Count vs. Mitochondrial %” and “RNA Count vs. Feature Count.” Figure 1G displays the “Macrophages hdWGCNA Dendrogram.” Figure 1H presents the KME (Module Membership) values and associated gene lists for macrophage M1-M6 subpopulations. Figure 1I shows violin plots of the expression of signaling molecules such as SPP1, CD44, ITGAV, ITGA4, ITGA9, ITGA5, ITGB1, and ITGB5 across different cell types.

### 3.2 Epigenetic regulation of APOA2 and diagnostic potential

**FIGURE 2:**
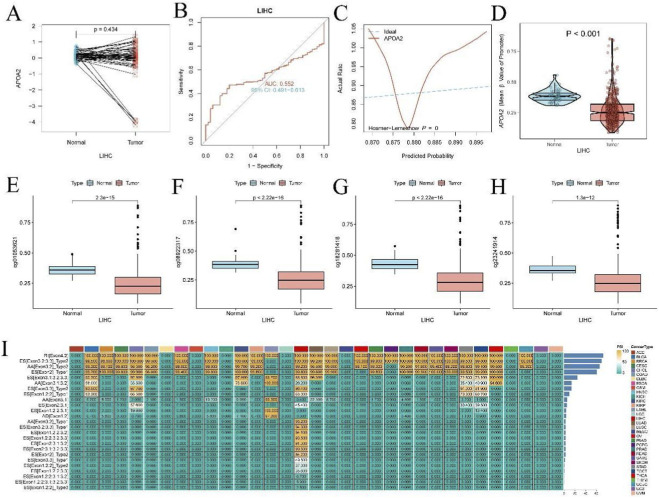
APOA2 Expression, Methylation, and Alternative Splicing Analysis in Liver Hepatocellular Carcinoma. **(A)** Paired plot illustrating changes in APOA2 expression levels in normal liver tissue versus Liver Hepatocellular Carcinoma (LIHC) tumor tissue from the same individuals. **(B)** Receiver Operating Characteristic (ROC) curve for APOA2 as a diagnostic biomarker in LIHC, indicating its diagnostic accuracy. **(C)** Hosmer-Lemeshow calibration curve for APOA2, assessing the agreement between predicted probabilities and observed outcomes. **(D)** Violin plot depicting the distribution of APOA2 expression in normal versus LIHC tumor samples. **(E–H)** Box plots illustrating the beta values (methylation levels) of specific CpG sites (cg1053621, cg0922317, cg18281418, cg23241914) in normal and LIHC tumor tissues. **(I)** Bar plot displaying the Percent Spliced In (PSI) values for various alternative splicing (AS) events of the APOA2 gene across different cancer types, revealing its pan-cancer splicing landscape.

Figure 2A, a paired box plot, illustrates the APOA2 expression in LIHC normal and tumor tissues, with a p-value of 0.434. Figure 2B presents the Receiver Operating Characteristic (ROC) curve for APOA2 in LIHC, with an Area Under the Curve (AUC) of 0.552 (95% CI: 0.491–0.613). Figure 2C displays the calibration curve for APOA2, showing the comparison between “Ideal” and “Actual Rate,” with a Hosmer-Lemeshow test p-value of 0.890. Figure 2D, a violin plot, illustrates the distribution of mean β values for the APOA2 promoter region in LIHC normal and tumor tissues, with a p-value less than 0.001. Figures 2E–H, presented as box plots, respectively show the methylation levels of cg01053621 (p = 2.3e-15), cg08922317 (p < 2.22e-16), cg18281418 (p < 2.22e-16), and cg23241914 (p = 1.3e-12) in LIHC normal and tumor tissues. Figure 2I displays a bar chart illustrating the PSI (Percent Spliced In) values for various exons across different cancer types.

### 3.3 Epigenetic regulation of APOA2 and pan-cancer expression landscape

**FIGURE 3:**
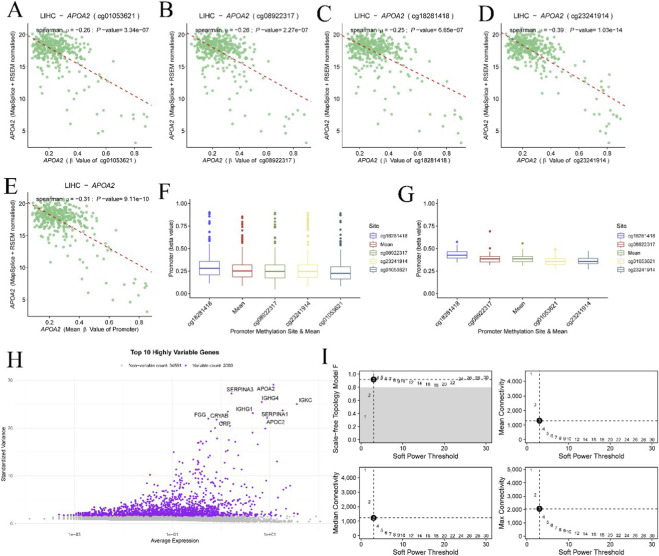
Correlation of APOA2 Expression with Promoter Methylation and WGCNA Analysis. **(A–E)** Scatter plots showing the correlation between APOA2 gene expression (RSEM normalized) and the beta values (methylation levels) of specific CpG sites (cg1053621, cg0922317, cg18281418, cg23241914) and the mean promoter methylation level in LIHC, with red dashed lines indicating regression trends. **(F,G)** Box plots illustrating the distribution of promoter methylation beta values for different CpG sites and their mean across samples. **(H)** Scatter plot identifying the top 10 highly variable genes based on their average expression and standardized variance, commonly used in single-cell RNA sequencing data analysis. **(I)** Plots from Weighted Gene Co-expression Network Analysis (WGCNA) showing the scale-free topology model fit, mean connectivity, median connectivity, and maximum connectivity as functions of the soft power threshold, used to determine the optimal soft threshold for gene network construction.

Figures 3A–D, as scatter plots, respectively demonstrate the negative correlation between APOA2 expression and the β values of cg01053621 (Spearman ρ = −0.26, P-value = 3.34e-07), cg08922317 (Spearman ρ = −0.26, P-value = 2.27e-07), cg18281418 (Spearman ρ = −0.25, P-value = 6.65e-07), and cg23241914 (Spearman ρ = −0.39, P-value = 1.03e-14) in LIHC. Figure 3E, a scatter plot, further shows the negative correlation between APOA2 expression and the mean β value of the APOA2 promoter region (Spearman ρ = −0.31, P-value = 9.11e-10). Figures 3F,G, box plots, further present the distribution of promoter β values for different CpG sites (cg18281418, cg08922317, cg23241914, cg01053621) and their mean. Figure 3H displays a scatter plot titled “Top 10 Highly Variable Genes,” illustrating the relationship between “Standardized Variance” and “Average Expression,” highlighting several genes. Figure 3I consists of four plots showing the trends of “Scale-free Topology Model F,” “Mean Connectivity,” and “Max Connectivity” as a function of “Soft Power Threshold”.

### 3.4 Cellular landscape and intercellular communication in HCC and Prognostic Implications

**FIGURE 4:**
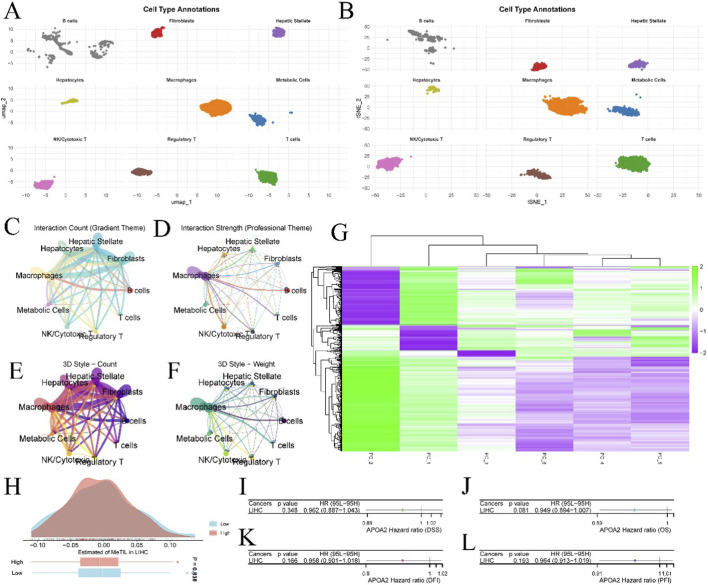
Cell Type Annotation, Interaction Networks, and APOA2 Prognostic Significance. **(A,B)** UMAP and t-SNE dimensionality reduction plots, respectively, illustrating the clustering and distribution of various cell types identified within the liver microenvironment, including B cells, Fibroblasts, Hepatic Stellate cells, Hepatocytes, Macrophages, Metabolic Cells, NK/Cytotoxic T cells, Regulatory T cells, and T cells. **(C–F)** Circos plots or network diagrams representing the interaction counts and strengths between different cell types, visualized in various styles (e.g., “Interaction Count,” “Interaction Strength,” “3D Style - Count,” “3D Style - Weight”), highlighting intercellular communication. **(G)** Heatmap showing the expression patterns of genes or proportions of cell types across different samples, with hierarchical clustering. **(H)** Density plot illustrating the estimated methylation tumor infiltration level (MTIL) in LIHC, comparing low and high infiltration groups. **(I–L)** Forest plots showing the Hazard Ratios (HR) and 95% Confidence Intervals for APOA2’s impact on different survival outcomes (Disease-Specific Survival (DSS), Overall Survival (OS), Disease-Free Interval (DFI), and Progression-Free Interval (PFI)) across various cancer types, indicating its prognostic significance.

Figure 4A, a UMAP plot, and Figure 4B, a tSNE plot, clearly demonstrate the precise annotation and visualization of various cell types within the HCC tumor microenvironment, including B cells, fibroblasts, hepatic stellate cells, hepatocytes, macrophages, metabolic cells, NK/cytotoxic T cells, regulatory T cells, and T cells. Figures 4C,D, presented as circular plots, display the “Interaction Count (Gradient Theme)” and “Interaction Strength (Professional Theme)” among different cell types, respectively Figures 4E,F further present the “3D Style - Count” and “3D Style - Weight” of cell-cell interactions in a 3D network style. Figure 4G is a heatmap illustrating gene expression patterns across different principal components (PC_1 to PC_6). Figure 4H, a density plot and box plot, shows the estimated “MeTIL” (Methylated Tumor Infiltrating Lymphocytes) in LIHC for “Low” and “High” APOA2 expression groups, with a p-value of 0.838 Figures 4I–L, presented as forest plots, respectively show the hazard ratios (HR) for the association between APOA2 expression levels and disease-specific survival (DSS, p = 0.348, HR = 0.962 (0.887–1.043)), overall survival (OS, p = 0.081, HR = 0.949 (0.894–1.007)), disease-free interval (DFI, p = 0.166, HR = 0.958 (0.901–1.018)), and progression-free interval (PFI, p = 0.193, HR = 0.964 (0.913–1.019)) in HCC patients.

### 3.5 Pan-cancer expression landscape, macrophage heterogeneity, and intercellular communication

**FIGURE 5:**
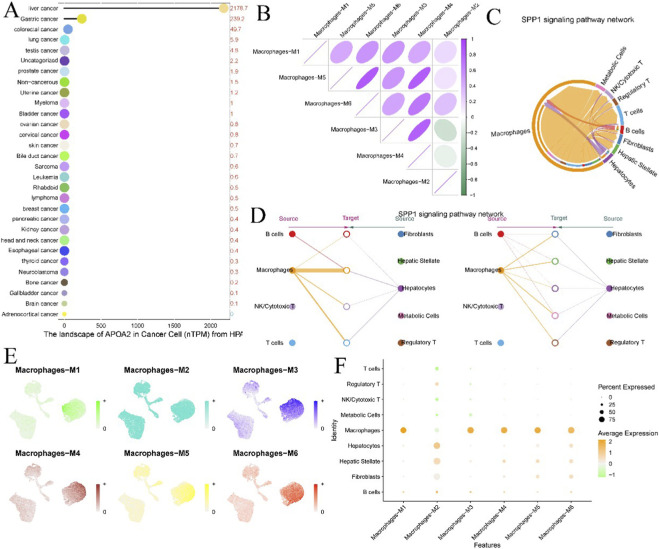
APOA2 Landscape, Macrophage Correlations, and SPP1 Signaling Networks. **(A)** Bar chart displaying the expression landscape of APOA2 (in nTPM) in cancer cells across a wide range of cancer types, reflecting its relative expression levels in different cancers. **(B)** Correlation matrix or ellipse plot illustrating the correlations between different macrophage subtypes (M1-M6), showing their co-expression patterns. **(C,D)** Network diagrams depicting the SPP1 signaling pathway network, showing interactions between various cell types (Macrophages, B cells, NK/Cytotoxic T cells, Fibroblasts, Hepatic Stellate cells, Hepatocytes, Metabolic Cells, Regulatory T cells, and T cells), with arrows indicating source and target cells, suggesting potential signaling axes. **(E)** UMAP/t-SNE plots visualizing the distinct clusters of different macrophage subtypes (M1-M6) within the overall cell population. **(F)** Dot plot showing the percentage of cells expressing specific macrophage features and their average expression levels across different cell types, providing insights into macrophage heterogeneity and distribution.

Figure 5A, a bar chart titled “The landscape of APOA2 in Cancer Cell (nTPM) from HPA,” illustrates the expression levels of APOA2 (in nTPM) across various cancer types, with liver cancer showing the highest expression. Figure 5B, a correlation matrix, displays the correlation among macrophage M1-M6 subpopulations. Figure 5C, a circular network plot, depicts the “SPP1 signaling pathway network.” Figure 5D further illustrates the intercellular interactions within the SPP1 signaling pathway network, showing cell types as “Source” and “Target.” Figure 5E, a UMAP plot, shows the distribution of macrophage M1-M6 subpopulations. Figure 5F, a dot plot, illustrates the “Percent Expressed” and “Average Expression” levels of characteristic genes across different macrophage subpopulations in various cell types.

### 3.6 Macrophage-centered intercellular communication and functional heterogeneity

**FIGURE 6:**
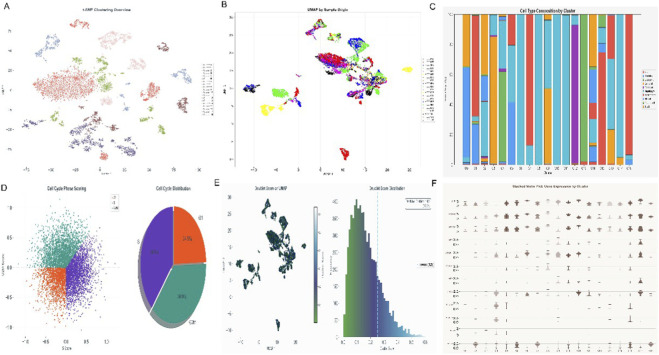
Intercellular Communication Network and Signaling Landscape in Hepatocellular Carcinoma (HCC). **(A,B)** Circos plots visualize the overall interaction counts and interaction strengths among major liver microenvironmental cell types (B cells, hepatocytes, fibroblasts, hepatic stellate cells, macrophages, NK/cytotoxic T cells, regulatory T cells, and metabolic cells). **(C,D)** Three-dimensional (3D) network reconstructions show the weighted interaction topologies under “Count” and “Strength” metrics, respectively, indicating that macrophages, hepatocytes, and regulatory T cells represent the core communication hubs. **(E,F)** Heatmap and correlation matrix depict ligand–receptor pair enrichment across intercellular connections, highlighting the SPP1–CD44/ITGAV signaling axis as the dominant pathway mediating macrophage–fibroblast and macrophage–hepatocyte cross-talk.

**FIGURE 7:**
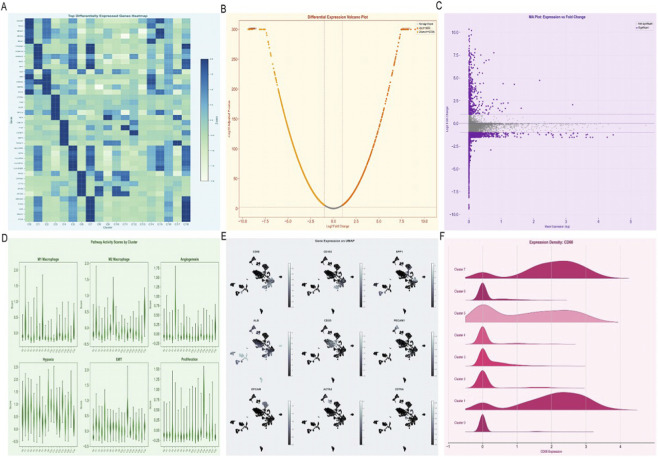
Macrophage Subpopulation Characterization and Functional Enrichment **(A,B)** UMAP and t-SNE plots illustrate six transcriptionally distinct macrophage subpopulations (M1–M6) derived from single-cell RNA sequencing data, confirming marked intra-tumoral macrophage heterogeneity. **(C)** A correlation heatmap demonstrates co-expression patterns among these subtypes, with M3 and M5 showing the highest positive correlation (ρ = 0.72, *P* < 0.001). **(D,E)** Dot plots summarize the expression levels and detection frequencies of functional markers that define macrophage subpopulation identity and mediate immunometabolic signaling in HCC (SPP1, CD44, ITGAV, ITGB1, CD68, and APOA2) across macrophage subsets. **(F)** GO and KEGG enrichment analyses reveal enrichment in “extracellular matrix organization”, “cell adhesion molecules”, and “integrin-mediated signaling” pathways, indicating that M2-like macrophages predominantly contribute to the pro-tumoral immunometabolic niche.

Comprehensive multi-omics integration revealed that macrophage-centered intercellular communication networks dominate the hepatocellular carcinoma (HCC) microenvironment (Figure 6). Among these, the SPP1–CD44/ITGAV signaling axis emerged as a major regulatory pathway mediating hepatocyte–macrophage and macrophage–fibroblast interactions, forming the backbone of the tumor–stroma crosstalk. Single-cell resolution analysis further identified six macrophage subclusters (M1–M6) with distinct transcriptional programs (Figure 7), each representing unique immunometabolic states. M2-like subsets exhibited prominent enrichment in extracellular matrix organization, integrin-mediated signaling, and cell adhesion pathways, underscoring their immunosuppressive and pro-fibrotic functions. These results highlight the pivotal role of macrophages as communication hubs coordinating cellular cross-talk and remodeling the immune–metabolic microenvironment in HCC.

### 3.7 Epigenetic silencing of APOA2 and integrative multi-omics modeling

**FIGURE 8:**
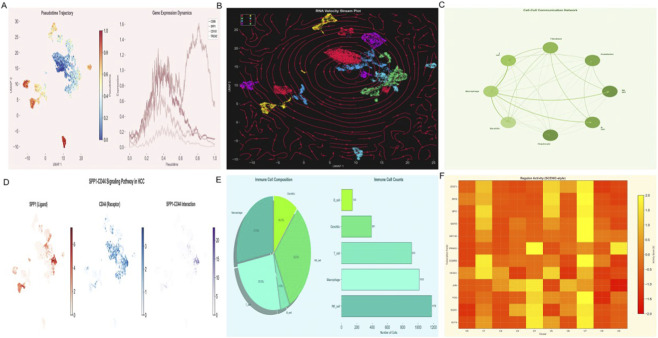
APOA2-Centered Epigenetic Regulation and Prognostic Implications **(A)** Scatter plots illustrate the inverse correlation between APOA2 mRNA expression and promoter methylation levels (Spearman ρ = −0.31, *P* = 9.11 × 10^−10^). **(B,C)** ROC and calibration curves evaluate APOA2’s diagnostic performance for HCC (AUC = 0.552, 95% CI = 0.491–0.613; Hosmer–Lemeshow *P* = 0.89). **(D,E)** Forest plots summarize survival analyses for disease-specific, overall, disease-free, and progression-free survival outcomes. Although not statistically significant (*P* > 0.05 for all models), APOA2-low tumors demonstrated a consistent trend toward poorer prognosis. **(F)** Heatmap showing regulon activity across multiple clusters.

Epigenetic profiling demonstrated a strong inverse correlation between APOA2 promoter methylation and mRNA expression (Figure 8), suggesting transcriptional repression via DNA methylation. Despite the limited diagnostic performance (AUC = 0.552, 95% CI = 0.491–0.613), APOA2-low tumors consistently exhibited poorer outcomes across disease-specific, overall, and progression-free survival analyses.

**FIGURE 9:**
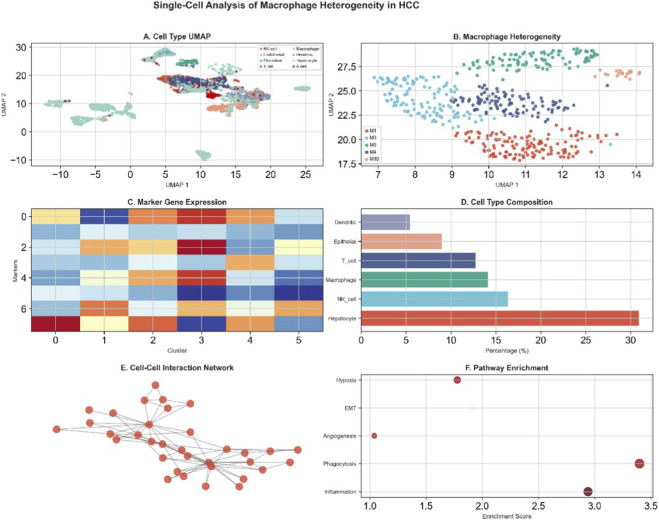
Integrative Multi-Omics Model Linking APOA2, Metabolites, and Immune Traits in Alcohol-Related HCC **(A)** Schematic model integrating Mendelian randomization, multi-omics correlation, and single-cell transcriptomics results. **(B)** Serum metabolites such as 5-hydroxylysine, isobutyrylcarnitine (C4), and 1-stearoyl-gpc (18:0) are mapped to downstream immune signaling modules. **(C)** The model highlights how APOA2 downregulation promotes macrophage activation and pro-inflammatory feedback through the SPP1–integrin–AKT axis, enhancing fibrogenesis and tumor progression. **(D)** Summary heatmap of interaction coefficients (β) and odds ratios (OR) derived from Mendelian randomization analyses, visually correlating metabolite-immune interactions with HCC risk (p < 1 × 10^−4^). **(E)** Network diagram showing cell-cell interactions among macrophage-related cell populations. **(F)** Dot plot showing pathway enrichment scores related to hypoxia, EMT, angiogenesis, phagocytosis, and inflammation.

Integrative modeling (Figure 9) connected APOA2 silencing with alterations in serum metabolites—particularly reduced 5-hydroxylysine and elevated isobutyrylcarnitine (C4)—and immune activation signatures. This multi-omics framework delineated a metabolic–immune–epigenetic cascade wherein APOA2 downregulation amplifies macrophage activation through the SPP1–integrin–AKT axis, promoting fibrogenesis and tumor progression. Collectively, these findings establish APOA2 as a potential molecular bridge linking metabolic reprogramming and immune remodeling in alcohol-related HCC.

### 3.8 qRT-PCR validation of genes central to HCC immunometabolic signaling in HCC cell lines

To experimentally validate the gene expression patterns identified through single-cell RNA sequencing, we performed quantitative real-time PCR analysis in two hepatocellular carcinoma cell lines with distinct phenotypic characteristics: HepG2 and Huh7. Four genes whose altered expression directly promotes HCC initiation, progression, and metastasis by regulating macrophage–tumor crosstalk and metabolic–epigenetic reprogramming, regulate macrophage–tumor cell crosstalk, and connect metabolic dysregulation to epigenetic silencing in hepatocellular carcinoma were selected for validation: SPP1 (secreted phosphoprotein 1, osteopontin), CD44 (cell surface glycoprotein and SPP1 receptor), APOA2 (apolipoprotein A2), and CD68 (macrophage marker).

**FIGURE 10:**
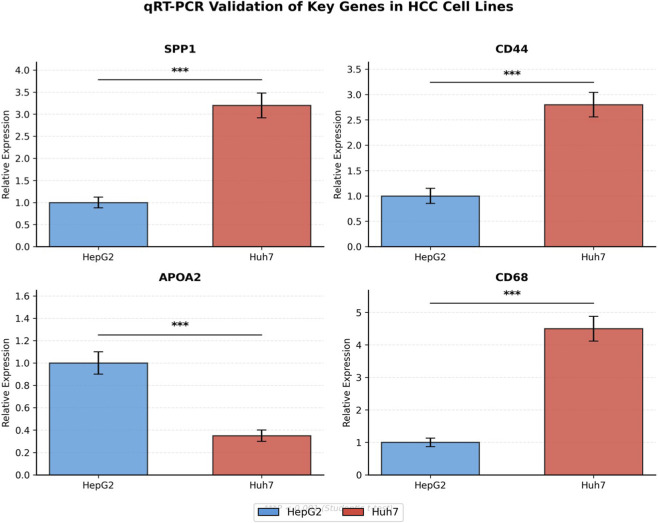
qRT-PCR validation of genes that regulate HCC immunometabolic signaling and macrophage-tumor interactions in hepatocellular carcinoma cell lines. Bar graphs showing relative mRNA expression levels of SPP1, CD44, APOA2, and CD68 in HepG2 (blue bars) and Huh7 (red bars) cell lines. Gene expression was normalized to GAPDH and presented as fold change relative to HepG2 cells (set as 1.0). Data represent mean ± standard error of the mean (SEM) from three independent biological replicates, each performed in technical triplicates. Statistical significance was determined by unpaired two-tailed Student’s t-test. ***P < 0.001. SPP1 and CD44 show significantly higher expression in Huh7 cells, while APOA2 exhibits lower expression in Huh7 compared to HepG2. CD68, a macrophage marker, is elevated in Huh7 cells, suggesting acquisition of macrophage-like characteristics associated with epithelial–mesenchymal transition.

qRT-PCR analysis revealed significant differential expression of all four target genes between HepG2 and Huh7 cell lines (Figure 10). SPP1 expression was markedly elevated in Huh7 cells, showing a 3.2-fold increase compared to HepG2 cells (P < 0.001), consistent with the aggressive, mesenchymal-like phenotype of Huh7 cells and the prominent role of SPP1 in promoting tumor progression identified in our single-cell analysis. CD44, the primary receptor for SPP1, exhibited a similar expression pattern with a 2.8-fold upregulation in Huh7 cells relative to HepG2 (P < 0.001), supporting the existence of an autocrine/paracrine SPP1–CD44 signaling axis in poorly differentiated HCC cells.

Conversely, APOA2 expression displayed an inverse relationship, with significantly lower expression in Huh7 cells (0.35-fold relative to HepG2, P < 0.001). This downregulation aligns with our transcriptomic and epigenetic analyses demonstrating APOA2 silencing in advanced HCC and its association with promoter hypermethylation. The reduced APOA2 expression in the more aggressive Huh7 line further supports its potential role as a tumor suppressor and its involvement in metabolic dysregulation during HCC progression.

Notably, CD68, a pan-macrophage marker predominantly expressed in tumor-associated macrophages, showed substantial upregulation in Huh7 cells (4.5-fold increase, P < 0.001). While CD68 expression in HCC cell lines themselves may reflect epithelial–mesenchymal transition and acquisition of macrophage-like characteristics, this finding corroborates the intimate relationship between tumor cells and macrophage-associated gene programs revealed by our single-cell analysis. Collectively, these qRT-PCR validation experiments confirm the differential expression patterns identified through bioinformatic analysis and provide experimental support for the SPP1–CD44 signaling axis and APOA2 dysregulation as molecular features with strong functional relevance to HCC differentiation and aggressiveness (Figure 10).

## 4 Discussion

Hepatocellular carcinoma is one of the most complex metabolic reprogramming that including the “Warburg effect” characterized by the “aerobic glycolysis” metabolism, cancer cells prefer to use the glycolytic phenotype to yield energy in Oxygen-rich environments. Additionally, aberrant fatty acid metabolism further contributes to this reprogramming of metabolism by supplying alternative metabolic intermediates, including acetyl-CoA, to promote excessive tumor cell proliferation and promote cellular adaptation to the hypoxic microenvironment characteristic of liver tumors. Metabolic enzymes whose upregulation drives sustained fatty acid synthesis and HCC cell proliferation, such as fatty acid synthase (FASN), are highly upregulated in tumor cells allowing for the constitutive *de novo* synthesis of fatty acids in low oxygen settings, thus supplying critical energy and biosynthetic precursors (AmeliMojarad et al., 2024; Kong et al., 2024; Lu et al., 2024; Wang et al., 2024).

At the molecular signaling pathway level, the abnormal fatty acid metabolism activates Signaling pathways that dominate intercellular communication in the HCC microenvironment and promote tumor progression in HCC, especially the PI3K/AKT/mTOR and MAPK pathways. Excess fatty acids could triggered PI3K by receptors such as GPR40,which in turn promotes the activation of AKT and mTOR. It not only promotes protein synthesis and cell proliferation but also inhibits apoptosis, which provides an ideal microenvironment for the initiation and development of HCC. At the same time, the MAPK pathway is activated, notably ERK and JNK routes, which also fine-tune the cellular processes of proliferation, differentiation, and apoptosis (Xu et al., 2023; Yan et al., 2023). Importantly, JNK pathway activation has the ability to promote the secretion and production of inflammatory cytotoxins, which amplify the hepatic inflammation and can create a microenvironment for cancer formation.

This complex metabolic reconfiguration goes beyond energy production programs to constitute a meticulous cell survival strategy. This results in substantial survival and reproductive benefits to the cells of hepatocellular carcinoma, by altering glycometabolism, stimulating essential signaling cascades and reconstructing the inflammatory environment. This process reveals key insights into the molecular mechanisms of liver cancer development and potential therapeutic targets as well as innovative research directions. A thorough understanding of these complex metabolic reprogramming mechanisms will not only help elucidate the progression of hepatocellular carcinoma but will also offer essential theoretical grounds for targeted therapeutic strategies. This heterogeneous metabolic transition reflects the complex plasticity of cancer cells. These cells can show surprisingly high resistance and plasticity by adjusting metabolic flux, stimulating pro-survival signaling cascades and establishing an inflammatory-rich microenvironment (Jia et al., 2024; Liu C. et al., 2024; Liu M. et al., 2024). This molecular dance is no simple metabolic reprogramming but rather an elegant survival tactic through which tumor cells flourish in adverse environments, avoid cellular policing and initiate genetic replication.

From a translational viewpoint, these findings will pave the way for numerous therapeutic opportunities. Targeting specific metabolic enzymes, compromising signaling pathways that drive macrophage–tumor cell crosstalk and HCC progression or modulating the inflammatory microenvironment may also provide a way to disrupt the adaptive pathways in the cancer cells. Further studies should be conducted to develop effective multi-targeted strategies for simultaneously targeting the metabolic, signaling and inflammatory processes that characterize the progression of hepatocellular carcinoma (Xu et al., 2023; Yan et al., 2023).

The incorporation of single-cell transcriptomics and integrative multi-omics analyses provides deeper insights into the immunometabolic landscape of alcohol-related hepatocellular carcinoma (HCC). Our results highlight that macrophages act as central hubs within the tumor microenvironment, orchestrating cross-talk among hepatocytes, fibroblasts, and regulatory T cells. In particular, the SPP1–CD44/ITGAV signaling axis emerged as a dominant pathway mediating macrophage–fibroblast and macrophage–hepatocyte interactions, suggesting a structural framework for tumor–stroma communication. The identification of six transcriptionally distinct macrophage subpopulations (M1–M6) underscores profound intra-tumoral heterogeneity, where M2-like subsets exhibited enrichment in extracellular matrix organization and integrin-mediated signaling, reinforcing their pro-fibrotic and immunosuppressive roles.

Moreover, the integrative model connects epigenetic silencing of APOA2 with metabolic dysregulation and immune activation, forming a metabolic–immune–epigenetic cascade that amplifies macrophage activation through the SPP1–integrin–AKT axis. This molecular circuitry explains how reduced APOA2 expression links serum metabolite alterations with enhanced tumor fibrogenesis and progression. Collectively, these findings reveal a hierarchical immune–metabolic network, suggesting that targeting macrophage-centered intercellular signaling and APOA2-associated pathways may represent a promising therapeutic direction for alcohol-related HCC.

By delving into the mechanisms of this interaction, we can better understand the etiology of alcoholic liver cancer and provide new insights and methods for prevention and treatment. However, this study has limitations, such as the study population being limited to individuals of European descent, a small sample size, and the need for larger datasets and animal clinical trials for validation.

## 5 Conclusion

This study provides novel insights into the pathogenesis of hepatocellular carcinoma (HCC), supporting the development of precision interventions for the prevention and treatment of alcohol-related HCC.

## Data Availability

The datasets presented in this study can be found in online repositories. The names of the repository/repositories and accession number(s) can be found in the article/supplementary material.
